# Repeatability of dental plaque quantitation by light induced fluorescence technology in current, former, and never smokers

**DOI:** 10.1186/s12903-023-03154-0

**Published:** 2023-07-13

**Authors:** Gianluca Conte, Amaliya Amaliya, Shipra Gupta, Rosalia Emma, Stefan Gospodaru, Gheorghe Bordeniuc, Valeriu Fala, Sebastiano Antonio Pacino, Salvatore Urso, Giovanni Zucchelli, Riccardo Polosa

**Affiliations:** 1 Department of General Surgery and Medical-Surgical Specialties, University of Catania, Catania, Italy; 2 Addendo Srl, Dental Clinic, Catania, Italy; 3 Department of Periodontology, Faculty of Dentistry, Universitas Padjadjaran, Bandung, West Java Indonesia; 4 Unit of Periodontics, Oral Health Sciences Centre, Post Graduate Institute of Medical Education and Research (PGIMER), Chandigarh, India; 5 Department of Biomedical and Biotechnological Sciences, University of Catania, Catania, Italy; 6 Fala Dental, Chișinău, Republic of Moldova; 7 “Nicolae Testemiţanu” State University of Medicine and Pharmacy, Chişinău, Republic of Moldova; 8 ECLAT Srl, Spin-off of the University of Catania, Catania, Italy; 9 Department of Biological, Geological and Environmental Sciences, University of Catania, Catania, Italy; 10 Department of Biomedical and Neuromotor Sciences, University of Bologna, Bologna, Italy; 11 Center of Excellence for the Acceleration of HArm Reduction (CoEHAR), University of Catania, Catania, Italy; 12 Department of Clinical and Experimental Medicine, University of Catania, Catania, Italy

**Keywords:** Smoking, Smoking cessation, Dental plaque, Dental calculus, Quantitative light-induced fluorescence, Reproducibility

## Abstract

**Background:**

The effects of smoking on the accumulation of dental plaque have not been studied in depth. We compared dental plaque quantitation obtained with a novel light induced fluorescence technology among current, former, and never smokers and verified measurements’ repeatability.

**Methods:**

Dental plaque quantitation was objectively assessed by quantitative light induced fluorescence (QLF) technology on three separate study visits in current, former, and never smokers: baseline (day 0), day 7, day 30. Increase in the fluorescence intensity of at least 30% (ΔR30) and 120% (ΔR120) together with the simple oral hygiene (SOH) scoring were considered for analysis.

**Results:**

The QLF parameters were highly repeatable in each study group (*p* < 0.0001, by regression analyses). All QLF parameters showed a significant difference between never smokers and current smokers (*p* = 0.041 for ΔR30; *p* = 0.027 for ΔR120; *p* = 0.04 for SOH). No significant differences were observed between never and former smokers and between current and former smokers except for ΔR120 (*p* = 0.033).

**Conclusion:**

Dental plaque measurements by QLF technology were highly reproducible and showed greater plaque formation among current smokers compared to non-smokers. Objective and reproducible quantitation of dental plaque can be a valuable clinical and regulatory science endpoint to investigate the effect of smoking cessation medications, combustion-free tobacco products, and consumer care products on oral health.

**Clinical relevance:**

There is a need to objectively evaluate the relationship between smoking and plaque build-up as well as maturation*.* Current smokers demonstrated greater and more mature plaque buildup when compared to never and former smokers. Differences in plaque build-up and maturation between current, former and non-smokers may be utilized as an effective tool for patient motivation, identifying therapeutic end-points, translational research as well as prognostication.

**Trial Registration:**

The study is a pilot study parts of a larger project with registration ID: NCT04649645. As preliminary study, the pilot study referred into this paper started before the larger study registered in ClinicalTrials.gov.

## Background

Dental plaque is an oral biofilm (i.e. mass of bacteria) that grows on the tooth surface, typically along the gingival line, or below the gingival line margins [1]. Build-up and maturation of dental plaque gives rise to dental calculi, which can be removed using dental scaling [2]. Poor oral hygiene (i.e., not brushing teeth thoroughly and regularly) is typically associated with dental plaque formation and build-up. Loe et al. [3] were the first to propose a pathogenetic role for bacteria in dental plaque for the onset of periodontal inflammation, and their research established the link between dental plaque and periodontal diseases. Therefore, one of the best methods for preventing periodontal disease is the routine removal of the dental plaque [4]. Local and systemic risk factors for dental plaque build-up and periodontal disease are well established [5–15]. Moreover, there is now a significant body of evidence to support significant independent associations between periodontitis and cardiovascular disease [16] and it is likely that reducing periodontal disease will have an overall positive impact on cardiovascular health.

The harmful health effects of smoking are well known, with a number of illnesses and conditions affecting much of the body's organs and systems [17, 18]. Cigarette smoking is also known to be a significant risk factor for the accumulation of dental plaque and the consequent periodontal disease [19, 20].

Although dental plaque scores are generally worse in tobacco cigarette smokers compared to non-smokers, accurate quantitation of dental plaque accumulation in people who smoke is lacking. Existing semiquantitative plaque scores have been shown to be poorly sensitive to discriminate differences between current, former and never smokers. For example, in the study by Lie et al. [21], smokers and non-smokers experienced similar rates of plaque formation. The lack of difference in dental plaque formation may be attributed to Silness and Löe plaque index's poor discriminatory power, which results from the subjective component of investigator scoring. The current study is designed to fill these gaps as it is related to a new technology that offered greater dental plaque discriminatory power to differentiate active smokers from non-smokers.

Cutting-edge approaches for the objective quantitation of dental plaque may improve the quality of oral health endpoints in clinical trials. Digital imaging methods can be used to measure dental plaque after prior use of plaque disclosing tablets or solutions. The first automated method based on digital imaging of methylene blue disclosed plaque was developed by Carter et al. [22]. The method had a definite advantage over the semiquantitative evaluation of dental plaque indices (e.g. plaque scoring requires trained assessors, can be subjective, has poor sensitivity due to the ordinal nature of the data), but it lacked careful standardization and the process was very time-consuming. Quantitative light-induced fluorescence (QLF) has been recently used to accurately measure dental plaque [23–26]. QLF measures dental plaque by quantitatively analyzing the red fluorescence from dental plaque induced by blue visible light at a wavelength of 405 nm, so it does not require any additional disclosing procedures.

There is paucity of literature on dental plaque changes in smokers after smoking cessation. We hypothesize that accurate quantitation of dental plaque is expected to show higher plaque accumulation in active smokers compared to non-smokers. Aim of this study was to compare dental plaque measurements as obtained by a novel QLF technology (QRayCam TM Pro, Inspektor Research Systems BV Amsterdam, NL) between current, former and never smokers. As a secondary aim of the study, we set out to verify repeatability of such measurements both in the short term (7-days interval) and in the long term (30-days interval) in order to determine the performance of the technology. Confirmation of good reproducibility of QLF measurements would give greater confidence about the strength of this technology for regulatory research applications when investigating the impact of consumer care products, smoking cessation drugs, and combustion-free tobacco products (e-cigarettes, heated tobacco products, oral tobacco/nicotine products) on oral hygiene.

## Methods

The methods have been described in more details in our previous published work [27].

### Study population

The study population consisted of three study groups identified among a pool of subjects who attended a smoking cessation clinic of the local university hospital in the previous 2 years, or contacted among hospital staff.

Study group 1 consisted of current smokers, defined as regular smokers of > 10 cigarettes per day for at least 3 years, with an exhaled carbon monoxide (eCO) level of ≥ 7 ppm.

Study group 2 comprised former smokers, defined as quitters who have been abstinent for no less than 1 year at the time of enrollment, with an eCO level of < 7 ppm.

Study group 3 consisted of never smokers, defined as subjects who never smoked or who reported having smoked less than 100 cigarettes in their lifetime [28]. Their eCO had to be < 7 ppm to exclude subjects significantly exposed to cigarette smoke or to environmental sources of carbon monoxide.

Current, former and never smokers had to satisfy the following inclusion criteria:

- 1. adult subjects (age 18–50 yrs).
- 2. absence of systemic diseases that may interfere with measurements.
- 3. presence of at least 10 natural anterior teeth (cuspid to cuspid, lower and upper jaw), with no prosthetics or crown.

Furthermore, they had to satisfy the following exclusion criteria (any conditions that could interfere with dental plaque measurements):

- 1. recent (less than 30 days) history of oral/dental infection/inflammation.
- 2. recent (less than 15 days) course of antibiotics or anti-inflammatory drugs.
- 3. subjects wearing fixed or removable orthodontic appliance or prothesis (limited to the 12 natural anterior teeth).
- 4. having undergone dental professional cleaning (i.e. scale and polish) within 6 months prior to screening.
- 5. pregnancy.

The study was conducted according to the Principles of Good Clinical Practice (GCP) and Declaration of Helsinki. The local Ethics Committee reviewed and approved the study.

### Study design

**Fig. 1:**
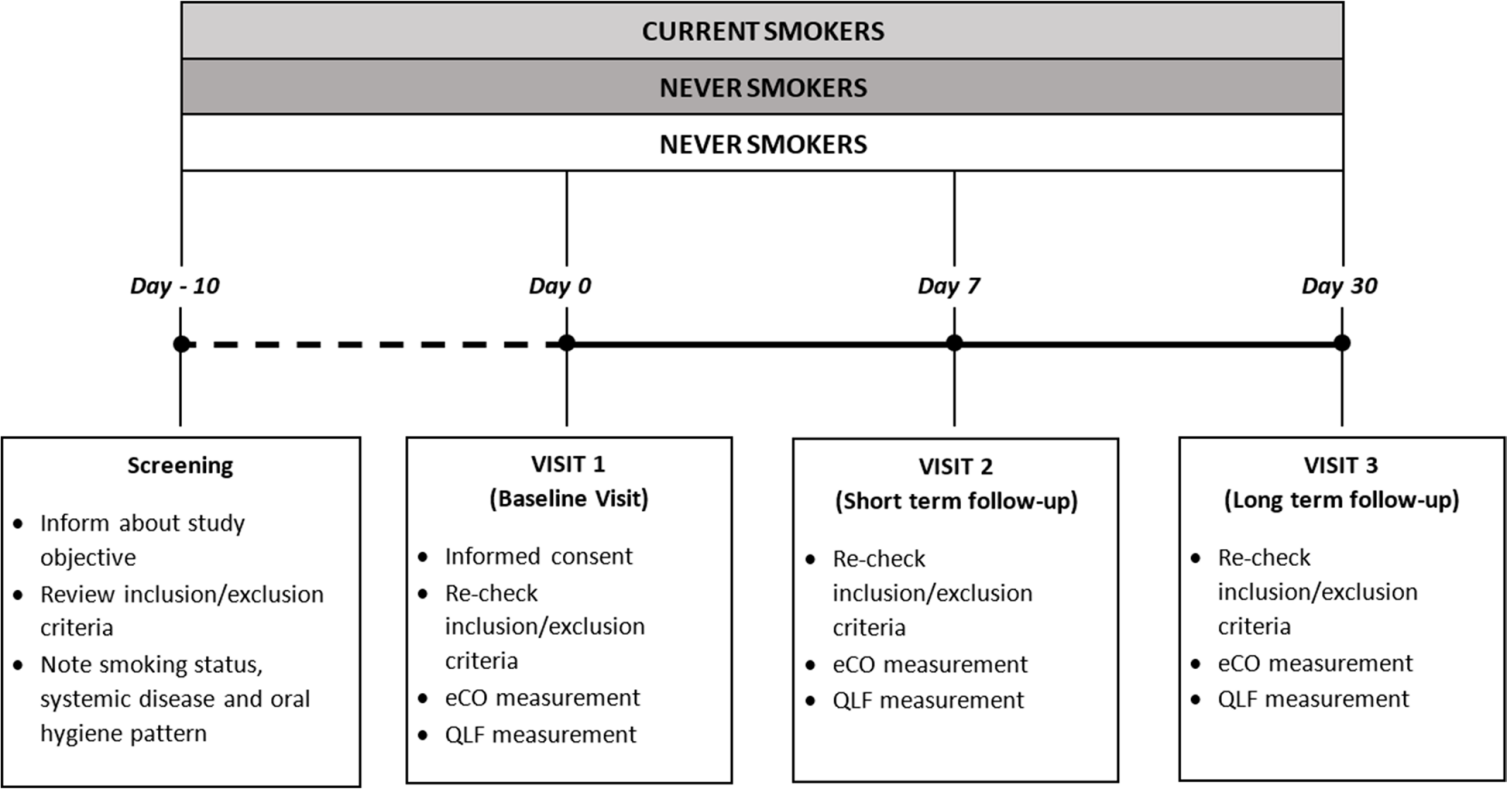
Schematic diagram of the study design

This is an observational study to compare dental plaque assessments by QLF technology between current, former, and never smokers and to assess their repeatability. The study consisted of a total of four visits: screening visit, baseline visit at day 0 (Visit 1), short-term follow-up visit at day 7 (± 1 days) (Visit 2) and long-term follow-up visit at day 30 (± 3 days) (Visit 3) (Fig. 1).

Participants were asked:

- not to change their habitual oral hygiene (toothbrushing, mouth washing, interdental-flossing) pattern for the whole duration of the study

- to avoid scaling and polishing procedures for the whole duration of the study
- not to floss for at least 72 h prior to each study visit
- not to smoke for at least 2 h prior to each study visit
- not to toothbrush and/or mouth rinse for at least 2 h prior to each study visit
- not to eat and/or drink (except water) for at least 2 h prior to each study visit

### Study Visits

#### Screening Visit

Potential participants attended a screening visit to (1) receive information about the rationale and objectives of the research; (2) verify their eligibility criteria; (3) assess smoking status and oral hygiene habit (i.e., frequency of toothbrushing, type of toothpaste, etc.); and (4) record general socio-demographic characteristics (i.e., sex, age, and occupation). All eligible subjects were invited to participate to Baseline Visit (Visit 1).

#### Baseline Visit (Visit 1)

This visit was carried out within 14 days of the Screening Visit. After re-checking inclusion/exclusion criteria and reviewing study restrictions, eCO measurement and QLF assessment were carried out, and baseline data was recorded. Subjects were instructed not to change their habitual oral hygiene pattern and invited to attend the next study visit (Visit 2).

#### Day-7 Visit (Visit 2)

Visit 2 was carried out within 10 (± 1) days after Visit 1. Eligibility criteria were verified again. QLF assessment was repeated for short term repeatability. Subjects were instructed not to change their habitual oral hygiene pattern and invited to attend the next study visit (Visit 3).

#### Day-30 Visit (Visit3)

Visit 3 was carried out within 30 (± 3) days after Visit 1. After re-checking eligibility criteria, QLF assessment was repeated for long term repeatability.

#### Exhaled Carbon Monoxide Measurement

Smoking status was objectively verified by measuring exhaled carbon monoxide (eCO) levels (eCO > 7 ppm indicating smoking status) with a portable CO monitor (Micro CO; Micro Medical Ltd, UK). Subjects were asked not to smoke cigarettes for at least 2 h before eCO measurements. Subjects were invited to exhale slowly into a disposable mouthpiece attached to the eCO monitor per the manufacturer's recommendations. The value of eCO readings was noted.

#### QLF Assessment

Prior to QLF assessment, subjects were asked to rinse their mouth with water and subjected to gentle flushing and drying by triple syringe tip to remove any food debris. Excess saliva was removed using a dental suction. Cheek retractors (Henry Schein, Gillingham, UK, Doubleend large, 106–7079) were placed to allow a good view of the 12 natural anterior teeth.

**Fig. 2:**
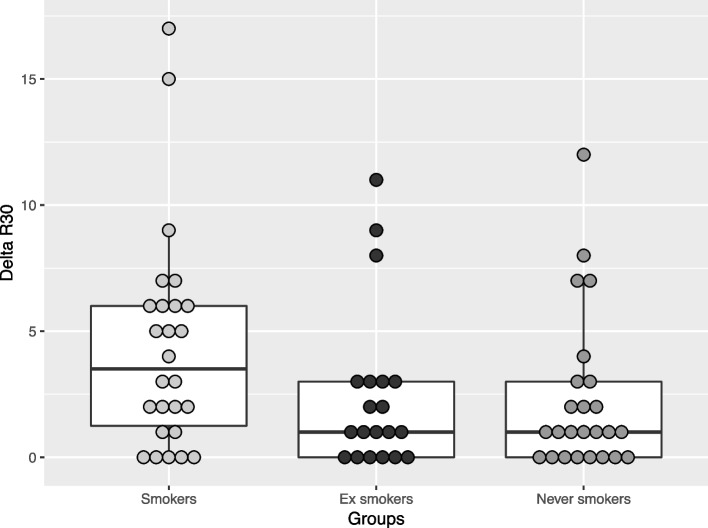
Boxplot illustration of Delta R30 comparisons among current smokers, ex-smokers and never smokers. Each dot represents the individual values of Delta R30 measurements. The median Delta R30 (illustrated by the bold line) was higher in Current Smokers, compared to other study groups

**Fig. 3:**
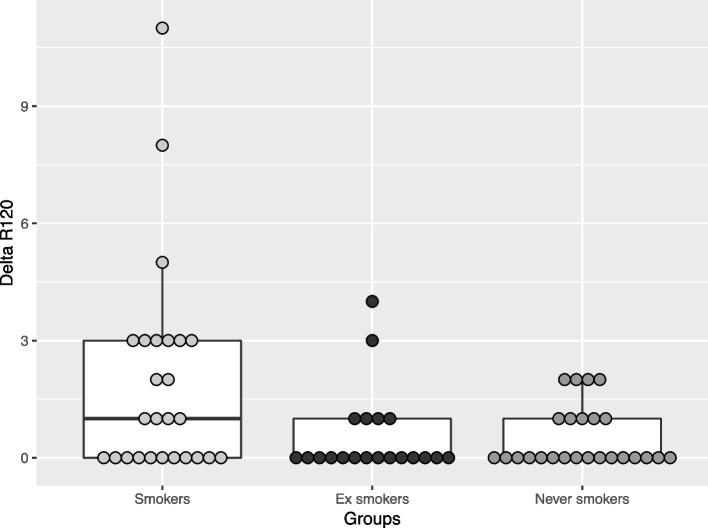
Boxplot illustration of Delta R120 comparisons among current smokers, ex-smokers and never smokers. Each dot represents the individual values of Delta R120 measurements. The median Delta R120 (illustrated by the bold line) was higher in Current Smokers, compared to other study groups

The Q-ray cam™ Pro (Co., AIOBIO, Seoul, Republic of Korea) is a high resolution, lightweight, handheld and auto-focus QLF™ camera. Images of the anterior teeth were taken with the Q-ray cam™ Pro according to the standard QLF digital photography protocol (see Fig. 2). The camera was brought as close to the subject’s teeth as physically possible (see Fig. 3). Ambient light level was standardized, making sure that any excessive ambient light is avoided. The auto-focus function of the camera was used to focus on the maxillary lateral incisor and canine (focal depth 0.32) and a white-light and a QLF image was taken automatically in quick succession and recorded by the Q-Ray™ software (QA v.1.41, Inspektor Research Systems BV, Amsterdam, NL). Q-ray cam™ Pro was used with the following settings: Resolution (image size), full high-definition [1920*1080 pixels]; shutter speed, auto [1/30–1/30000 s]; aperture, auto [F1.2–360]; sensor object distance, 2.3 Mpixel Image Sensor. Images were taken by three different investigators who had been trained by the manufacturer.

Fluorescence photographs of the vestibular aspect of the anterior teeth (cuspid to cuspid, upper and lower jaw) in end-to-end position were taken. Images were automatically saved by default as a bitmap image using a QLF proprietary software (QA v.1.41, Inspektor Research Systems BV, Amsterdam, NL). The software was used to analyze the images to determine, for each pixel on the dental surface, the value of ΔR which is a measure of the increase of red fluorescence relative to the sound surface. Higher ΔR values indicate areas with more active bacterial metabolism within the dental plaque, representing a greater level of dental plaque maturation. Moreover, the rationale for assigning ΔR30 and ΔR120 as the key measurements has been explained previously in Park et al. [26]. In brief, fluorescent plaque index (FPI) is a plaque scoring system that takes the area as well as the thickness of plaque into account. Traditional plaque scoring methods assess only area or thickness, but not both. Low FPI scores (0–2) are determined by the low intensity red fluorescent plaque areas (A30), while high scores (3–5) are determined more by high intensity red fluorescent plaque areas (A120). Since the fluorescence intensity of the red fluorescent plaque is related to plaque thickness, dental plaque with higher red fluorescence intensity (ΔR120) can be considered indicative of thicker plaque than plaque with a relatively low red fluorescence intensity (ΔR30).

Therefore, the percentage of the total tooth surface showing an increase in the fluorescence intensity of at least 30% (ΔR30) indicates the total area of mature dental plaque detected, whereas an increase in the intensity of at least of 120% (ΔR120) reveals only areas of greater level of plaque thickness/maturation (i.e. calculus/tartar) within the total area of mature dental plaque detected. The QLF proprietary software (QA v.1.41, Inspektor Research Systems BV, Amsterdam, NL) also integrated the fluorescence intensities data to calculate the simple oral hygiene (SOH) scoring [26]. SOH scoring may also estimate the degree of gingival inflammation.

## Data analysis

This is a proof-of-concept pilot study, the first of its kind, hence no previous data could be used for power calculation. Please note that our study related to a new technology that offered theoretically greater discriminatory power than existing semiquantitative scores but which, to date, has not been evaluated in significant human populations. Meaningful power calculation requires an a priori estimate of the performance of the technology – the estimation of which is also a key objective of the current study. In this context, any power calculation would be based on little more than assumptions and consequently would not be an informative metric for the study design.

Normal distribution of the data was assessed using Kolmogorov–Smirnov test. Counts and percentages summarized categorical data; median (interquartile range; IQR) were used to summarize continuously distributed data with skewed distribution. Data comparisons among study groups were carried out by Chi-square test for categorical data and Kruskal–Wallis test for continuously skewed data.

Repeatability of QLF parameters (ΔR30, ΔR120, SOH) were evaluated by linear regression analysis of measurements obtained at V1 vs. V2 (short-term repeatability) and at V1 vs. V3 (long-term repeatability) for each study group. Moreover, the mean differences and the limits of agreement (95% confidence interval) were calculated to assess the agreement between V1 vs. V2 and V1 vs. V3 for each study group. A 1-tailed sample Wilcoxon signed rank test was also performed to assess the mean difference between two measurements from zero. Finally, to evaluate the intra-rater reliability of the intrasession measurements among the three visits (V1, V2, and V3), the intra-class correlation coefficient (ICC) was computed using a single-measurement, absolute-agreement, two-way mixed-effect model [29].

Multiple regression analysis was performed to assess the interaction of age, gender, cig/day (only for smokers group), years of smoking (only for smokers group), year of non-smoking (only for ex-smokers group) and oral hygiene parameters on test results.

All analyses were considered significant with a *P* value < 0.05. R version 3.4.3 (2017–11-30) was utilized for data analysis and generation of graphs.

## Results

### Study participants

A total of 71 subjects (27 F; median age of 33 years) completed the study: 26 current, 20 former, and 25 never smokers (Table 1). No significant differences were observed among the three study groups at baseline, with the exception of weekly frequency of mouthwashing.

**Table 1:** Clinical characteristic of study groups

	**Smokers**	**Ex- Smokers**	**Never Smokers**	***p*** ** value**
**Subjects, n**	26	20	25	
**Age (yr)**	30.5 (26–38.25)	35.5 (25–38.5)	34 (29.5–37)	0.313
**Female**	8/26 (30.8%)	8/20 (40%)	11/25 (44%)	0.610
**Frequency toothbrushing (daily)**	2 (1.6–3)	2.5 (2–3)	2 (2–3)	0.260
**Frequency mouthwashing (per week)**	0 (0–0)	0 (0–1)	1 (0–2)	0.002
**Frequency dental floss usage (per week)**	0 (0–1.4)	1 (0–1.5)	1 (0–1.5)	0.760
**No. Cig./Day**	15 (12–19.5)	//	//	NA
**Year smoking**	11 (8.5–19.75)	//	//	NA
**Pack/years**	9.75 (5.6–12.5)	//	//	NA
**Year non-smoking**	//	2.5 (1.5–7.65)	//	NA

Data are presented as median (IQR), n/N (%) unless otherwise stated

*NA* Not applicable

### Repeatability of Dental Plaque Quantitation in never smokers

Results of short-term (7 days) and long-term (30 days) repeatability analyses in never smokers are summarized in Table 2. Significant linear regressions were observed for all QLF parameters (ΔR30, ΔR120, and SOH scores) both for short-term (study visit 2 vs. study visit 1) and long-term (study visit 3 vs. study visit 1) repeatability assessments, with R squared values being always > 0.86. Moreover, all the means of the differences for QLF parameters among the visit 1, 2, and 3 were not significantly different from zero.

**Table 2:** Dental QLF parameters repeatability analysis in Never Smokers

Parameters	Short-Term Repeatability			Long-Term Repeatability		
	Regression analysis V2-V1 R value (*p* value)	Mean of the difference V2-V1 being different from zero?		Regression analysis V3-V1 R value (*p* value)	Mean of the difference V3-V1 being different from zero?	
		Mean of the Difference (95% CI)	YES/NO (*p* value)		Mean of the Difference (95% CI)	YES/NO (*p* value)
ΔR30	0.951 (*p* < 0.0001)	0.16 (-1.19/1.51)	NO (*p* = 0.256)	0.864 (*p* < 0.0001)	0.08 (-2.32/2.48)	NO (*p* = 0.746)
ΔR120	0.924 (*p* < 0.0001)	-0.12 (-077/0.53)	NO (*p* = 0.083)	0.901 (*p* < 0.0001)	-0.08 (-0.62/0.46)	NO (*p* = 0.162)
SOH score	0.975 (*p* < 0.0001)	0.08 (-0.46/0.62)	NO (*p* = 0.162)	0.920 (*p* < 0.0001)	0 (-0.98/0.98)	NO (*p* = 0.425)

*CI* Confidence interval, *V1* Visit 1, *V2* Visit 2, *V3* Visit 3

### Repeatability of Dental Plaque Quantitation in current smokers

Results of short-term and long-term repeatability analyses in current smokers are summarized in Table 3. All QLF parameters (ΔR30, ΔR120, and SOH scores) showed significant regressions analyses both for short-term (V2 vs. V1) and long-term (V3 vs. V1) repeatability assessments, with R squared values being always > 0.97. Moreover, all the means of the differences between V1 and V2, and between V1 and V3 for QLF parameters were not significantly different from zero, with the only exception of the mean difference for ΔR30 in the long-term repeatability assessment (mean diff. = -0.31; *p* = 0.015).

**Table 3:** Dental QLF parameters repeatability analysis in Smokers

Parameters	Short-Term Repeatability			Long-Term Repeatability		
	Regression analysis V2-V1 R value (*p* value)	Mean of the difference V2-V1 being different from zero?		Regression analysis V3-V1 R value (*p* value)	Mean of the difference V3-V1 being different from zero?	
		Mean of the Difference (95% CI)	YES/NO (*p* value)		Mean of the Difference (95% CI)	YES/NO (*p* value)
ΔR30	0.971 (*p* < 0.0001)	-0.08 (-1.54/1.38)	NO (*p* = 0.627)	0.985 (*p* < 0.0001)	-0.31 (-1.38/0.77)	YES (*p* = 0.015)
ΔR120	0.990 (*p* < 0.0001)	-0.08 (-0.61/0.46)	NO (*p* = 0.346)	0.978 (*p* < 0.0001)	0 (-0.78/0.78)	NO (*p* = 1)
SOH score	0.978 (*p* < 0.0001)	0 (-0.55/0.55)	NO (*p* = 1)	0.974 (*p* < 0.0001)	-0.12 (-0.75/0.52)	NO (*p* = 0.149)

*CI* Confidence interval, *V1* Visit 1, *V2* Visit 2, *V3* Visit 3

### Repeatability of Dental Plaque Quantitation in former smokers

Results of short-term and long-term repeatability analyses in former smokers are summarized in Table 4. Both short-term (V2 vs. V1) and long-term (V3 vs. V1) repeatability assessments showed significant regressions analyses for all QLF parameters (ΔR30, ΔR120, and SOH scores), with R squared values being always > 0.91. Moreover, all the means of the differences between V1 and V2, and between V1 and V3 for QLF parameters were not significant different from zero.

**Table 4:** Dental QLF parameters repeatability analysis in Ex-Smokers

Parameters	Short-Term Repeatability			Long-Term Repeatability		
	Regression analysis V2-V1 R value (*p* value)	Mean of the difference V2-V1 being different from zero?		Regression analysis V3-V1 R value (*p* value)	Mean of the difference V3-V1 being different from zero?	
		Mean of the Difference (95% CI)	YES/NO (*p* value)		Mean of the Difference (95% CI)	YES/NO (*p* value)
ΔR30	0.966 (*p* < 0.0001)	0.05 (-1.14/1.24)	NO (*p* = 0.85)	0.925 (*p* < 0.0001)	0.15 (-1.57/1.87)	NO (*p* = 0.588)
ΔR120	0.962 (*p* < 0.0001)	-0.05 (-0.49/0.39)	NO (*p* = 1)	0.955 (*p* < 0.0001)	-0.05 (-0.82/0.72)	NO (*p* = 0.773)
SOH score	0.912 (*p* < 0.0001)	-0.05 (-1.05/0.95)	NO (*p* = 0.766)	0.948 (*p* < 0.0001)	0.05 (-0.72/0.82)	NO (*p* = 0.346)

*CI* Confidence interval, *V1* Visit 1, *V2* Visit 2, *V3* Visit 3

### Intra-Rater Reliability of Dental Plaque Quantitation

Intraclass correlation coefficients (ICCs) were calculated for each QLF parameter within each study group in order to assess both degree of correlation and agreement between intrasession measurements (Intra-rater reliability). The ICC values and their 95% confidence intervals are reported in Table 5. We observed that all the measurements of QLF parameters exhibited a marked level of reliability (values > 0.93) with significant *p* values (< 0.0001).

**Table 5:** Intra-rater reliability of QLF parameters within smokers, ex-smokers, and never smokers groups

Intraclass Correlation (ICC)		95% Confidence Interval		F Test With True Value 0			
		Lower bound	Upper bound	F Value	df1	df2	*p* value
**Smokers**							
ΔR30	0.986	0.973	0.993	223	25	47.9	< 0.0001
ΔR120	0.993	0.987	0.997	422	25	52	< 0.0001
SOH score	0.983	0.967	0.992	178	25	50.7	< 0.0001
**Ex-smokers**							
ΔR30	0.974	0.947	0.989	111	19	39.4	< 0.0001
ΔR120	0.955	0.909	0.98	62.2	19	38.8	< 0.0001
SOH score	0.970	0.939	0.987	96	19	39.7	< 0.0001
**Never smokers**							
ΔR30	0.941	0.889	0.971	47.4	24	49	< 0.0001
ΔR120	0.932	0.873	0.967	43.2	24	49	< 0.0001
SOH score	0.966	0.935	0.984	83.8	24	49.6	> 0.0001

ICC estimates and their 95% confident intervals were calculated based on a mean-rating (k = 3), absolute-agreement, 2-way mixed-effects model

### Comparisons of Dental Plaque Quantitation among current, former and never smokers

A small, but non-significant, difference was observed for Delta R30 score between the three study groups (*p* = 0.0765). The median (IQR) values were respectively 3.5 (1.25–6) in current smokers, 1 (0–3) in former smokers, and 1 (0–3) in never smokers (Fig. 2). Cross-comparisons between each study group showed a significant difference between never smokers and current smokers (*p* = 0.041), but no significant difference was observed between never smokers and former smokers (*p* = 0.787), and between current smokers and former smokers (*p* = 0.08).

For Delta R120 score, a significant difference among the three study groups was observed (*p* = 0.032). The median (IQR) values were respectively 1 (0–3) for current smokers, 0 (0–1) for former smokers, and 0 (0–1) for never smoker group (Fig. 3). Cross-comparisons between each study group showed significant differences between never smokers and current smokers (*p* = 0.027), and between former smokers and current smokers (*p* = 0.033). No significant difference was observed between never smokers and former smokers (*p* = 0.712).

**Fig. 4:**
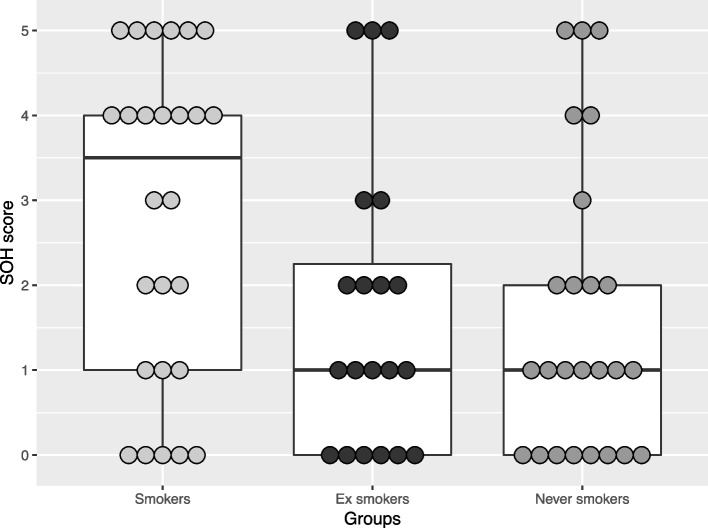
Boxplot illustration of SOH score comparisons among current smokers, ex-smokers and never smokers. Each dot represents the individual values of SOH scoring. The median SOH score (illustrated by the bold line) was higher in Current Smokers, compared to other study groups

A small, but non-significant, difference was observed for SOH score between the three study groups (*p* = 0.070). The median (IQR) values were respectively 3.5 (1–4) for current smokers, 1 (0–2.25) for former smokers, and 1 (0–2) for never smokers (Fig. 4). Cross-comparisons between each study group showed a significant difference between never smokers and current smokers (*p* = 0.04), but no significant difference was observed between never smokers and former smokers (*p* = 0.842), and between current smokers and former smokers (*p* = 0.068).

### Assessment of interaction effects on Dental Plaque Quantitation

Multiple regression analyses were performed to identify individual variables, including age, gender, cig/day (only for smokers group), years of smoking (only for smokers group), year of non-smoking (only for ex-smokers group), and oral hygiene parameters. which may influence measurements of QLF parameters.

None of the individual variables investigated had significant effect on the regression analyses, assessing the short-term repeatability and long-term repeatability of the three QLF parameters in the study groups.

No significant interaction was observed on assessment of comparisons among the study groups, with the exception of the covariate “frequency of toothbrushing (daily)”, which was significantly related to Delta R30 score (*p* = 0.0004), Delta R120 (*p* = 0.0004), and to SOH (*p* < 0.0001) scores on assessment of comparisons among the study groups.

## Discussion

This study investigated the impact of smoking and smoking abstinence on dental plaque quantitation by comparing measurements obtained with a novel QLF technology among current, former, and never smokers. Objective quantitation of dental plaque showed that oral health of current smokers is substantially inferior compared to non-smokers. Study findings also show high and consistent level of repeatability for QLF measurements.

Current smokers had consistently higher QLF measures of mature dental plaque (i.e. ΔR30), calculus (i.e. ΔR120) and simple oral hygiene (SOH) scores compared to non-smokers, indicating worse overall oral health due to chronic exposure to cigarette smoke. The findings of this paper are consistent with the notion that smoking can contribute to accumulation of dental plaque and the consequent risk of periodontal disease [19, 20]. Several studies have suggested that smokers had significantly more supragingival plaque than non-smokers and were prone to have more severe periodontitis [30–32]. The impact of smoking on the periodontium has been shown to be dose-dependent with both the duration and daily quantity of consumption affecting outcomes [33].

Although a clear difference in dental plaque, calculus, and SOH was observed between current smokers and never smokers, the discrepancy between current smokers and former smokers failed to reach statistical significance for dental plaque (*p* = 0.08) and SOH (*p* = 0.068). This might have been due to the smaller sample size of former smokers population and it is therefore likely that the observed trend could have become statistically significant with a larger sample size. An alternative explanation for the lack of significant difference between current and former smokers is that the relatively short duration of smoking abstinence (2.5 years on average) in our sample of former smokers was not long enough to allow further improvement of dental plaque parameters. It is also possible that high prevalence of periodontitis among former smokers could have influenced findings. Changes in smoking behavior alone are unlikely to affect the condition and reversal of periodontitis among smokers who quit may take up to 10 years to become significant [34]. As a consequence, it is desirable that clinical trials assessing changes of QLF endpoints should exclude or account for subjects with periodontitis. Intensity and frequency of personal oral hygiene (in particular toothbrushing) is another important confounder. Although we took the precaution of asking participants to avoid changing their personal oral hygiene habits throughout the duration of the study, multiple regression analyses showed that “frequency of toothbrushing (daily)” was the only covariate with significant interaction on Delta R30 (*p* = 0.0004), Delta R120 (*p* = 0.0004), and SOH (*p* < 0.0001) scores when assessing comparisons between study groups. This has clear implication for clinical research in oral health and more specifically for prospective assessment of dental plaque changes; 1) a standardized approach for personal oral hygiene must be always considered when designing oral health studies**;** 2) to reduce measurement variability and increase the size of detectable changes of QLF endpoints in prospective studies, baseline data must be collected after scaling and polishing. By eliminating dental plaque and calculus, the best possible oral health state is reached at the start of the study and changes can be meaningfully referenced to baseline data. This is a critical and innovative element for clinical trial design in oral health research.

Former smokers did not tend to differ significantly from never smokers in terms of levels of QLF measures of mature dental plaque (i.e. ΔR30), calculus (i.e. ΔR120) and simple oral hygiene (SOH) scores. Although these findings suggest the importance of smoking abstinence in determining oral health improvements, only prospective studies can provide definitive confirmation.

This is the first study to investigate repeatability of dental plaque quantitation by QLF technology in current, former, and never smokers. Study findings show significant short- and long-term repeatability in all study groups for all dental plaques indices. Test variability is a major concern when investigating dental/oral outcomes in clinical trials and the reported good reproducibility of QLF measurements in this study was reassuring.

A few factors and limitations need to be considered when interpreting these study findings. First, the study populations consisted of relatively young subjects, and their dental plaque measurements may not be representative of the general population. Consequently, additional studies with more representative age groups are needed to confirm our findings. Second, dental plaque measurements were performed only on the vestibular surface of the dental arch (cuspid to cuspid, upper and lower jaw). We limited our evaluation to anterior teeth because it is relatively easy to obtain a good view of the buccal aspect with one single photograph. Also while we acknowledge that posterior regions harbor larger amount of plaque than anterior regions, plaque scoring frequencies shows consistent relationship between anterior and posterior regions [35]. Therefore, it is unlikely that the interpretation of study findings would have changed significantly by extending measurements to all dental regions. Third, COVID-19 restrictions had only a minimal impact on the study conduct because it was carried out in between the first and second wave of the pandemic in Italy (July 2020-October 2020) when clear guidelines for dental settings were already set in place and most restrictions to dental clinic access were lifted.

## Conclusions

By adopting an innovative cutting edge technology for objective and consistent quantitation, we showed that smokers have more dental plaque compared to non-smokers. These findings may have important implications for smoking cessation. For those smokers who perceive bad breath and poor teeth appearance as a significant problem, improvements in dental plaque changes may be an important driver for giving up smoking. Moreover, objective, reproducible discrimination of dental shade measurements will increase confidence in their value for a range of applications, including clinical and regulatory research applied to combustion-free tobacco products (e.g., e-cigarettes, heated tobacco products, oral tobacco/nicotine products), smoking cessation medications, and consumer care product for oral hygiene and dental aesthetics.

## Data Availability

All data generated or analysed during this study are included in this published article.
